# Undertaking Community Engagement for a Controlled Human Malaria Infection Study in Kenya: Approaches and Lessons Learnt

**DOI:** 10.3389/fpubh.2022.793913

**Published:** 2022-04-29

**Authors:** Noni Mumba, Patricia Njuguna, Primus Chi, Vicki Marsh, Esther Awuor, Mainga Hamaluba, Cynthia Mauncho, Salim Mwalukore, Johnson Masha, Mary Mwangoma, Betty Kalama, Hassan Alphan, Juliana Wambua, Philip Bejon, Dorcas Kamuya, Melissa C. Kapulu

**Affiliations:** ^1^Kenya Medical Research Institute (KEMRI) Wellcome Trust Research Programme, Kilifi, Kenya; ^2^PATH Centre for Vaccine Innovation and Access, Nairobi, Kenya; ^3^Centre for Tropical Medicine and Global Health, Oxford University, Oxford, United Kingdom

**Keywords:** community engagement approaches, human infection studies, malaria, stakeholder identification, challenges and lessons

## Abstract

Human infection studies (HIS) involve deliberately infecting healthy volunteers with disease-causing pathogens under controlled conditions. These studies are “controlled” by way of using specific types of pathogens, including dose, and the availability of emergency medical facilities to research volunteers. Most HIS involve diseases whose treatment is known and are done to accelerate the development of novel therapeutics such as vaccines, to address emerging and existing infectious diseases. Traditionally, HIS have been conducted primarily in high-income countries (HICs) but are now increasingly being conducted in low-and-middle income countries (LMICs). In LMICs settings, HIS are likely to raise concerns among various stakeholders including participating populations and regulatory bodies, that are unfamiliar with this type of research. Deliberately infecting a healthy individual with a disease-causing pathogen seems to go against the normal practice of medicine of “do no harm”. Such types of studies can give rise to increased rumors and jeopardize research participation in study activities, including non-HIS research. Community engagement can be one approach to address particular issues that HIS studies raise through meaningfully engaging with communities, where views and voices inform the conduct of HIS studies. In addition, engagement can inform the ethical conduct and acceptability of HIS studies in LMICs settings and provide opportunities for sharing information, listening to, and responding to concerns and views from potential participants, and the larger community in which the study would be conducted. Despite community engagement being an important aspect to consider, very few published and gray literature cover the types of approaches that have been used, and lessons learnt in engagement for HIS. This article outlinesthe community engagement approaches that were used to engage stakeholders and communities for malaria HIS-controlled human malaria infection (CHMI), undertaken in Kilifi, Kenya. It outlines the engagement activities across the research cycle, from activities conducted during protocol development, to planning, and implementation of the study. We discuss the challenges experienced, lessons learnt, and provide some recommendations for engagement around HIS.

## Introduction

Human infection studies (HIS), otherwise known as controlled human infection studies, challenge studies, and human challenge trials, involve deliberate infection of healthy volunteers through administering pathogens under controlled conditions (1). Controlled conditions refer to the specificity of the pathogen, dose, close monitoring of research volunteers, and availability of emergency medical services. HIS are deemed to be cost-effective as they provide an opportunity for accelerated testing of vaccines to provide estimates of vaccine safety and efficacy (2). Such studies are conducted with the aim of: (i) evaluating candidate vaccines and other therapeutics; (ii) gaining insight into host responses in natural infections; and (iii) developing a model of infection (1).

Traditionally, HIS have been conducted primarily in high-income countries (HICs) but many target infectious diseases occurring mostly in low-and-middle-income countries (LMICs). Some of the reasons why this has been the case include limited (but rapidly growing) infrastructure, skills/training to undertake such studies, and inadequate legal, ethical, and regulatory systems in LMICs. Community understanding and acceptability of such studies can also be a reason why these studies have taken time to be conducted in LMICs settings. In recent years, capacity building initiatives targeting LMICs have significantly addressed some of these gaps, which has also contributed to an increasing number of HIS conducted in these settings (3).

While conducting HIS in LMICs is a welcome idea, these types of studies require careful development of research approaches that support both scientific and ethical conduct. The idea of deliberately infecting a healthy individual with a pathogen goes against the ethical norm in clinical practice and research of “do no harm” (4). Safety concerns, rumors, and misinformation can also undermine willingness to participate in study activities. Thus, community engagement can play a critical role in providing accurate information and opportunities for community members to interact with research and researchers and discuss concerns and how best these could be addressed. Engagement also provides forums to discuss consent, recruitment strategies, inconveniences arising from study participation, ancillary care that could be provided, and how to begin to address many of these issues including potential third-party risks. Importantly, community engagement can inform research teams whether it would be acceptable for HIS to be conducted in a particular community, and thus whether or not to continue with a planned HIS. However, as has been documented widely for other types of studies, community engagement in HIS presents several complexities including what approaches are appropriate to use, whom to engage, and competing goals of the engagement. Despite community engagement being an important aspect to consider, there is limited literature covering the types of engagement approaches that have been used for HIS in LMIC settings specifically, and the lessons being learnt.

## Community Engagement for HIS

The HIS can raise concerns among communities and the broader public if appropriate steps to engage communities are not taken. Such concerns can be around: the type of pathogen involved (including perceptions of the immediate and longer-term health and social implications of deliberate infection); the experience and implications of requirements for residency away from home during the study where this is a requirement; discomfort or health risks related to the study procedures (such as frequent blood sampling); perceptions around treatability of disease following deliberate infection (including possibilities of third party risks); and limits to the right to withdraw that may be in place to protect the volunteer (5). Therefore, the researchers need to plan appropriate stakeholder engagement to inform the study design and implementation; an engagement plan should consider who should be engaged and how to engage with the stakeholders right from inception stage of a HIS, through implementation until post-end of the study.

Several published works have demonstrated the importance of community and stakeholder engagement for HIS. In a recent study that assessed the acceptability of SARS-COV-2 HIS, conducted in the UK among 20 to 57-year-olds, volunteers suggested that due to the ethical complexities and public interest in such studies, it was important to ensure transparency to the public and broader scientific communities (6). Similarly, workshops conducted in India (7), Kenya (8), Malawi (9), Uganda (10), and Zambia (1) assessed acceptability of HIS for varying pathogens. Participants of these workshops included researchers from HICs and LMICs, community representatives (10), representatives of ministries of health, community and public health specialists, research funders, journalists, and lawyers. Relevant to some of these workshops was the pre-workshop consultation and engagement of community stakeholders including potential volunteers (1, 10).

In India, workshop participants identified important considerations for HIS, including the role of ethics review committees in safeguarding the rights of research volunteers, considerations of legal implications on deliberate infection of healthy people, and other social considerations such as engaging the media (7). Reviewing these critical aspects of HIS requires that ethics committees have a good understanding of the context within which such studies are conducted (11). Participants of a workshop in Malawi assessed the views of stakeholders on a pneumococcal carriage HIS and found that participants would be supportive of such studies provided stringent safety processes would be put in place and communities and stakeholders were appropriately engaged (9).

Studies have also shown the importance of community engagement for HIS in LMIC settings, especially among populations with either little research experience or where this type of study is implemented for the first time (8). Community engagement can provide early information and understanding in populations from which research volunteers may be drawn, thereby helping with the process of obtaining informed consent (4). Supporting principal investigators to spend time in and interact with communities where participants will be drawn from, including directly engaging with local residents, has been shown to strengthen trust and a sense of mutual respect and understanding (12).

Even though the importance of careful community and stakeholder engagement is emphasized in the literature, there is the little emphasis given to the approaches used or their value. Here, we aim to share our experiences, including challenges and lessons learnt during the development and implementation of engagement activities for malaria HIS conducted in Kilifi Kenya, to provide a resource for researchers and engagement practitioners in other LMIC contexts.

## A Program of HIS on Falciparum Malaria in Kenya

Over the last 6 years, a program of HIS on falciparum malaria involving over 160 volunteers has been conducted at the Kenya Medical Research Institute (KEMRI)-Wellcome Trust Research Programme (KWTRP) and Center for Geographic Medicine Research Coast (CGMR-C), in Kilifi (peri-urban and rural Kenya), under a program titled “Controlled Human Malaria Infection in Semi-Immune Kenyan Adults” (CHMI–SIKA) (13).

The CHMI–SIKA program of work in Kilifi followed an initial “proof of principle” HIS on falciparum malaria at the KEMRI Center for Clinical Research in Nairobi (urban area and capital city of Kenya), involving 28 healthy semi-immune adults, recruited mainly from medical colleges and those living within the vicinity of the research center in Nairobi in 2013 (14). Given the novelty of this research approach, study implementation was preceded by consultation and engagement with national-level science, ethics and medicines regulatory bodies, and universities within the vicinity of the research center, over a 2-year period (8). This initial and continuing engagement with very high-level stakeholders helped to pave way for the conduct of CHMI–SIKA in Kilifi.

## The Controlled Human Malaria Infection in Semi-Immune Kenyan Adults Study

The CHMI–SIKA study involved residents of low, moderate, and high malaria endemicity areas from the Coast and Western Kenya (15). The study aimed to better understand immunity to malaria with the potential to identify antigen targets that could be developed as second-generation malaria vaccine candidates. This study was set up as an open-label infectivity non-intervention study enrolling healthy Kenyan adults with varying exposure to malaria. A total of 161 healthy adult volunteers were enrolled and infected with *Plasmodium falciparum* (PfSPZ Challenge) sporozoites following successful recruitment and screening to ensure healthy status. These volunteers were admitted to a residential facility a day before infection and monitored for the development of any signs and symptoms of malaria. The study outcomes and procedures have been described previously (13, 15). In brief, the main aim of the study was to investigate how the *in vivo* parasite growth rate of *Plasmodium falciparum* is modified by pre-existing immunity measured by antibody levels to blood-stage antigens with the following objectives:

(a) Measure correlations between antibody levels to defined and well-characterized malaria antigens and growth rates of *P. falciparum* in volunteers undergoing CHMI.
(b) Confirm the safety of CHMI administered by direct venous inoculation in semi-immune volunteers.
(c) Measure parasite growth rates in semi-immune volunteers.
(d) Establish a sample set for the study of immunity to malaria and its effect on parasite growth following CHMI in semi-immune volunteers.
(e) Explore the understanding, motivations for participation, and experiences of volunteers and other stakeholders.

The implementation of this study in a different setting outside of the initial setting of Nairobi provided an opportunity for a context-specific undertaking of community engagement activities with a clearly outlined strategy to inform practice. This community case study focuses on local engagement approaches for the CHMI–SIKA study in Kilifi, which was made possible by the early buy-in of national-level stakeholders.

## The Study Site

The KWTRP has its headquarters in Kilifi, with 2 other research hubs in Nairobi and Mbale (Eastern Uganda). Kilifi County is one of the 47 Counties in the devolved government system of Kenya, located in the Coastal part of the country, bordering the Indian Ocean. It is a rural County, with fast-rising peri-urban towns. The residents of the county are predominantly from the Mijikenda community. The population of Kilifi has low-literacy levels, and the main economic activities include tourism, farming, and fishing.

**Figure 1:**
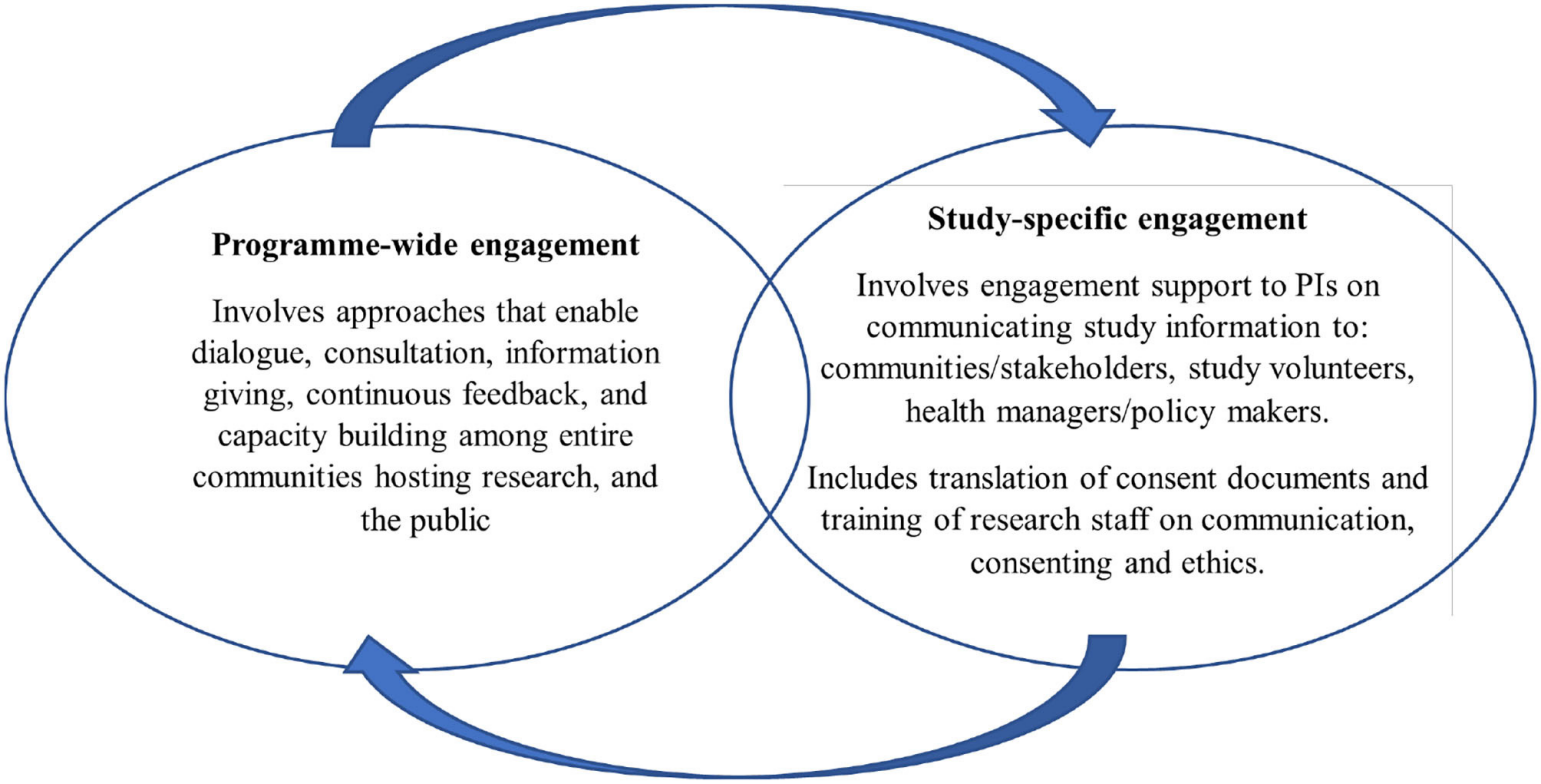
Programme-wide and study-specific community engagement at KWTRP.

The KWTRP Kilifi hub hosts a range of international and national collaborative research projects, including epidemiological, social, laboratory and clinical research, to inform local, national, and international health policy. The research activities at KWTRP are supported by a strategic community, public and policy engagement platform, with specific experienced engagement staff (Community Liaison Group, CLG) responsible for implementing engagement activities (16). The program also includes a Kilifi health and demographic surveillance system (KHDSS) of over a quarter million residents (17), from which research volunteers are drawn, for the studies conducted in Kilifi. The overall community engagement goal in Kilifi is building and sustaining mutual understanding and trust between research staff and host communities, in support of generating new knowledge on health. Strategically, engagement is structured around ongoing overarching “program-wide” activities, and activities focused on specific research projects, including the HIS conducted in Kilifi. Figure 1 above summarizes these components of the KWTRP engagement strategy.

Community engagement at the Kilifi hub of KWTRP is supported by a network of around 200 community representatives (KEMRI Community Representatives, KCRs), elected by residents living within the KHDSS (18). The KCRs are a hybrid community advisory board and serve a 3-year term, after which they retire, and new representatives are elected. Furthermore, engagement activities include open days at the Kilifi research center (including workshops targeting specific gatekeepers such as local registered self-help groups and religious leaders), an innovative schools engagement program, media engagement, and engagement with healthcare providers and managers, and policymakers in local and national departments of health.

## Planning and Implementing Community Engagement for CHMI–SIKA

### Planning

The CHMI–SIKA study began in 2016 with volunteer recruitment and was conducted over 3 years (2016–2018). Healthy adults aged between 18 and 45 years were injected with *P. falciparum* sporozoites and were required to be full-time residents in a study facility for up to 25 days for close clinical and research monitoring. Study procedures and stakeholder experiences are detailed in a series of clinical trial and social science publications (4, 19), including the CHMI–SIKA protocol (15).

**Figure 2:**
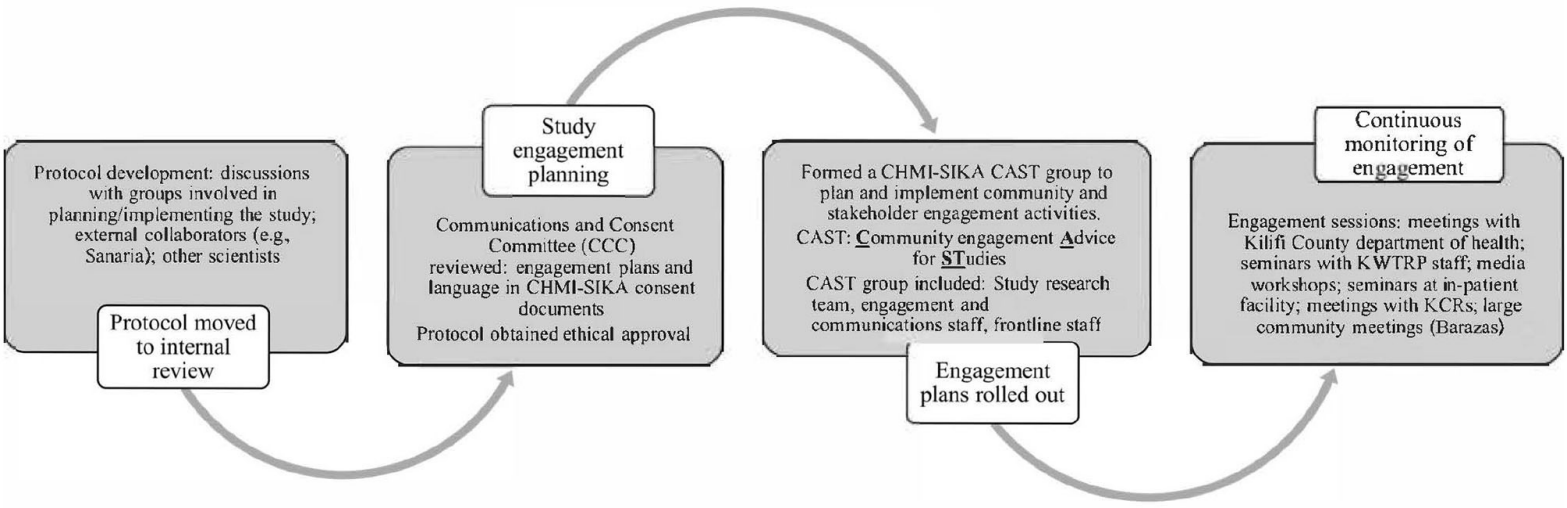
Flow diagram depicting how engagement planning was done for the CHMI–SIKA study.

Planning for community and stakeholder engagement for CHMI–SIKA began early, as part of the development and preparation of the study protocol for scientific and ethical approval. The flow chart in Figure 2 here demonstrates the stages for planning for engagement from protocol development, all through to implementation of study engagement activities.

As happens for all other studies, a CAST (Community engagement Advice for STudies) team was formed and deliberated on all aspects of community and stakeholder engagement throughout the planning, review, and implementation of the CHMI–SIKA study (20). The CAST is made up of representatives of the study such as the principal investigator, a study coordinator/clinician, members of the engagement team (CLG), and a social scientist (where relevant). In the implementation of CHMI–SIKA study engagement activities, information giving roles were split between one representative of the CLG, who handled generic research information and specific questions about KWTRP from the audience being engaged, and one or two members of the study team who handled specific study information, as outlined in the key messages document. For example, during engagements with community members, members of the CHMI–SIKA CAST who attended these sessions included: (i) a study investigator with a medical background who was able to respond to questions that were clinical; (ii) a field worker who explained specifics about mobilization and recruitment; (iii) a CLG staff whose main role was moderating the entire session from start to end, including responding to general questions about research and KWTRP functions. A CAST group can have up to 10 members, however about 3-5 members attend engagement events, with different members of the CAST group attending different engagement sessions based on their availability.

A critical first step for the CHMI–SIKA CAST team given the novel research approach in this setting was to identify and consider the implications of research features that were unique to this approach. This was an important step as it laid the foundation for the next steps which included mapping stakeholders and outlining approaches to be used for engagement, and the development of messages for engagement sessions. Unique features of CHMI–SIKA discussed at the initial CAST meeting that were considered sensitive and/or new in our context included: (i) healthy volunteers would be deliberately infected with malaria parasites; (ii) volunteers would be required to stay in full residency during a prolonged period (up to 25 days) during the study; and (iii) volunteers from Ahero, in Western Kenya (about 850 km from Kilifi) would travel to join their Kilifi counterparts participating in the study. This design ensured that healthy adults with a range of levels of prior malaria exposure were included since malaria has high endemicity in Western Kenya and low-to-moderate endemicity across Kilifi. Through their deliberations, the CAST team identified the stakeholders to be engaged and engagement approaches to be used (Table 1) and developed key messages that would ensure consistent and correct messaging during engagement for CHMI–SIKA. In developing key messages, the CAST members considered the unique features of this study and framed communication about the study based on these features. This meant that these features were specifically addressed in every engagement session, maintaining correct and consistent engagement messaging. Key messages also included other study procedures such as the amount of blood drawn in the study, which is still a sensitive issue in the Kilifi population (Table 2). The process outlined here is specific for Kilifi as a different engagement approach was undertaken in Ahero based on their prevailing stakeholder engagement activities.

**Table 1:** Key stakeholders identified by the CAST team for CHMI–SIKA in Kilifi and engagement approaches used.

**Stakeholder identified**	**Engagement approach used**
• *Local Department of Health:* as health gatekeepers in Kilifi County this group is charged with ensuring all research is relevant, safe and that volunteers are protected from harm. During the initiation of the CHMI-SIKA study, they provided the researchers with access to participating communities and have more recently evolved to provide formal approval for research studies to be conducted within their jurisdiction.	• *Meetings:* As with other non-HIS studies, the Head of Engagement requested for a slot in the agenda of a weekly county health management team (CHMT) meeting. The CHMT comprises of very senior health managers at County government level (21).
• *KEMRI Community Representatives (KCRs):* a network of local community members serving as a hybrid community advisory board (CAB) (18), and a link with local community members.	• *Meetings:* Conducted specific meetings with KCRs from locations where participants would be drawn from. •*Workshops:* CHMI-SIKA team presented the study during routine KCRs workshops held at KWTRP campus.
• *Chiefs, Assistant Chiefs and Village Elders:* administrative arm of the government at location, sub-location, and village level, responsible for oversight of all activities being implemented at that level.	• *Courtesy calls and meetings:* conducted meetings with administrators and village elders first, as these are the gatekeepers at community level.
• *KWTRP staff:* all staff whose job responsibilities bring them into contact with community members (such as field workers, drivers).	• *Seminars:* a series of seminars were conducted within KWTRP campus targeting all staff, but specifically those whose roles include direct interaction with the community (e.g., frontline staff)
• *Local University population:* the study inpatient facility was located within a local university.	• *Seminars:* conducted a series of seminar targeting students and faculty
• *Media/Journalists:* identified specific local and national media groups (mainly print editorial staff) for study awareness.	• *Media workshops/meetings:* A series of meetings were conducted between CHMI-SIKA investigators and specific journalists from Kilifi, Nairobi and internationally.
• *Study volunteers:* individuals already screened and admitted into in-patient facility	• *Open Days: workshop-like meetings which include a tour of KWTRP laboratories*
• *Community members:* local communities in study areas	• *Large meetings (Barazas):* with the assistance of chiefs, a series of barazas within the community were held.

**Table 2:** Key messages derived by the CAST team for community engagement in the CHMI–SIKA study.

**The key messages were framed around:**
• The question researchers wanted to answer with the study, and why it was important
• The study site(s), targeted study volunteers, and study procedures
• Risks/costs of study participation as well as potential benefits
• Safety issues in deliberately infecting healthy volunteers, certainty around the nature of what was being injected
• Health concerns over the possibility that treatment given eventually would fail to achieve a cure
• Safety issues in relation to the total volume of blood taken, given that sampling was to be frequent over a prolonged period of time
• Information around what would happen in the case of serious adverse events or death
• In-patient stay for around 25 days, and how volunteers would be compensated for their time away from employment/business.

### Implementation of Engagement Approaches

#### Stakeholder Meetings

The CHMI–SIKA study team were allocated time to present this study to Kilifi County department of health stakeholders during one of their routine County health management team (CHMT) meetings. The Head of Engagement worked with the coordinator of CHMT meetings to identify a suitable date and time, and then the study investigator was informed. A member of the research team (often the PI or study coordinator) gave a 10-min presentation, and then took questions, comments, and recommendations from the health managers, including discussion and approval of the strategies developed by the study CAST team.

#### Meetings With Community Representatives

Study sensitization meetings with members of a network of community representatives (KEMRI Community Representatives, KCRs) drawn from 3 locations where study volunteers were going to be recruited from (Junju, Banda-ra-Salama, and Ngerenya), were conducted. The community representatives shared their concerns as community members, and concerns that could come from those they represented in their respective villages. Giving them information about the CHMI–SIKA study and responding to their concerns, meant that they were better equipped to respond to questions from community members whom they represented.

#### Community Meetings (Barazas)

KWTRP has a well-established relationship with local area Chiefs, their assistants and village elders within the KHDSS. In Kenya, Chiefs form part of the national administrative arm of the government responsible for interior security. As such, part of their responsibility includes maintaining security at the community level and disseminating or enforcing relevant government policies within their localities. Chiefs are considered important gatekeepers in the community and approve activities that involve community members to happen at the community level. Important information is communicated to the general public through organizing community *barazas*, which are large meetings of community members. Chiefs are responsible for calling the *baraza*. Community members are mobilized from their homes by word of mouth, through village elders (these elders work under instruction from a Chief). Meetings cannot begin without the presence of a local Chief. During the meeting, the Chief makes opening remarks, before inviting guests to make their presentation to community members. At the end of the engagement meeting, it is again the responsibility of the Chief to close and disperse the audience. In some instances, the Chief may summarize his/her learning or understanding of the study as part of closing remarks. *Barazas* are typically attended by 100–200 community members if well mobilized and they usually take place during mid-morning hours. These meetings provided a forum for directly engaging community members on the CHMI-SIKA study. Between 70 and 150 community members from sub-locations of the 3 main locations named earlier were reached with CHMI-SIKA study messaging. The meetings began with a member of the CLG giving a general overview of KWTRP and research activities conducted by scientists at the center, and then a CHMI–SIKA study team member was invited to talk about the study. This was followed by a question-and-answer session.

Important gaps in a wider understanding of the research context were highlighted through more general questions asked about how KWTRP conducts research activities. For example, community members wanted to understand why KWTRP focuses mainly on certain diseases such as malaria, and not other common illnesses affecting the community such as filariasis or hypertension. These questions were responded to by a CLG staff and CHMI–SIKA study team present in the sessions who explained the process of arriving at a research question, including the review of hospital mortality data.

#### Seminars

From routine engagement activities, we have come to understand that KWTRP staff are important gatekeepers in the community as they are often asked many questions about the work of the Programme. To ensure that all the staff in the Programme were aware of this study and that any concerns/questions they had were addressed appropriately, the CLG staff organized a series of seminars where the CHMI–SIKA team presented the study and responded to questions that were raised (refer to Table 3).

**Table 3:** Common questions and concerns about CHMI–SIKA raised by community members, study volunteers, stakeholders and KWTRP staff who participated in engagement sessions.

• What if the required 18–45 age bracket people who will consent become less than the required number, can an over age person be recruited? (Community members and study volunteers)
• What happens when one dies after being injected with the malaria parasite? (Community members and study volunteers)
• Will you cater for the families of those you will ‘admit’ because they won't be able to work for their families? (Community members and study volunteers)
• If I come for the screening and you find out that I have a [health] condition, will you treat me? (Study volunteers)
• While ‘admitted’ at [local] in-patient facility, will I be allowed to go home to [visit] my family and then come back, or not? (Community members and study volunteers)
• What happens if after admission [being challenged and treated] I fall sick again? (Study volunteers)
• What is the possibility of non-clearance of parasites with antimalarials at the end of the study and what could be the effects of that on me? (Community members and study volunteers)
• What if I am a heavy drinker of alcohol? (Community members)
• How do you get the parasites? From people or from the mosquitoes? (Community members)
• Relationships can be affected if one partner consents to participate in this study, and the other refutes their partner's participation. (Community members, study volunteers, KWTRP staff)
• Why does KEMRI take a lot of blood from participants (also linked to devil worship)? (Community members, study volunteers)
• There is no privacy and confidentiality at the study in-patient facility; drawing of blood is done openly (in view of other volunteers). (Study volunteers)

*In parenthesis included are examples of stakeholder groups that raised the question(s)*.

#### Open Days for Research Volunteers During Residency

The study team came up with the idea of engaging the study volunteers, as a way of keeping them busy during their in-patient stay and improving their understanding of health research during residency. After administration of malaria parasites (between days 2 and 5 post-infection), the research volunteers had a workshop in-residency and then were invited into the research institution for a tour of the research facilities (e.g., laboratories where study samples were being processed and stored) and interaction with scientists. The study team also took this opportunity to further respond to questions from the volunteers, concerning the CHMI–SIKA study. Volunteers were picked in groups from the in-patient facility in a bus and immediately transported back after the engagement meeting. This was done to ensure that all volunteers got back to the in-patient facility without breaking study protocols and going home to visit family/friends. The open days also provided an opportunity for CLG staff to discuss with the study volunteers more broadly about KEMRI as an organization and provide a holistic view of the research conducted.

#### Media Workshops

The CHMI–SIKA study team participated in a media engagement workshop organized by KWTRP's communications team for researchers at the Programme. During the workshop, scientists shared a round table with one or two journalists and discussed with them their research areas of interest, including ongoing or planned work. Through this workshop, the study was explained to journalists who were present.

#### Across all Activities

An important feature of the engagement activities undertaken was that CHMI–SIKA study team members (principal investigator, study coordinator, lead clinician, and project manager) attended and participated in all the engagement sessions alongside experienced members of the community engagement team. When the scientists participate in engagement activities, they can hear first-hand, issues that are of concern to potential research volunteers. They are also able to learn about and consider social and cultural aspects that are important to the community where a study is being conducted (22). In addition, this allows for the community to have study-specific procedures thoroughly explained and provides a layer of information given prior to informed consent. Table 3 provides an example of concerns and questions that were raised during the various CHMI–SIKA engagement sessions.

## Challenges, Lessons Learnt, and Recommendations

The CHMI–SIKA research study was the first of its kind in Kilifi. It was also the first time that the study volunteers were drawn from different parts of the country and put together in one site. Thus, the experience of the CLG staff in systematic planning for research studies helped us prepare for this unique study. At first, having a structure such as the CAST group that is set up for every study involving human subjects was important as it aided in carefully thinking through important points of consideration for engagement, using an engagement template. The engagement template has a section for sensitivities in a study; here, unique features of CHMI–SIKA were listed. From this list, key messages were developed to aid in correct and consistent messaging. Secondly, going out into the community gave potential volunteers an opportunity to (i) meet the CHMI–SIKA team, (ii) hear first-hand about the study, and (iii) have their concerns about the study responded to. Having researchers directly interact with community members and discuss planned research is one way to build respectful relations and provide opportunities to discuss areas that worry the community as was the case with CHMI–SIKA and can contribute to building trust. Finally, using a combination of approaches ensured that many different stakeholders were reached with engagement activities and had opportunities to have their concerns responded to. Systematic engagement is very involving and time-consuming; thereby requiring ample planning time so as not to interfere with study timelines.

At the end of every engagement session, conducting what the CLG calls “debrief meetings” in all engagement activities helped to review what worked well and what did not. For every CHMI–SIKA engagement activity, the engagement team met to discuss and formulate strategies for improving what did not work well. Emerging new concerns not captured in earlier developed key messages were shared with the study team and responses fed back to the stakeholder or community group engaged. Such meetings are helpful as the implementing teams can review what works and what does not work well, and how challenges faced can be mitigated in future engagement sessions. To support learnings in engagement, embedding empirical social science work within ongoing HIS has built a better understanding of study benefits and risks (5), and highlighted critical engagement aspects that may be overlooked in the course of activity planning and implementation. The engagement strategy at KWTRP is deliberately linked to social science so as to ensure that there is a continued loop of implementing, evaluating, learning, and adapting/changing.

The social science team was able to draw on some of the similarities and differences in engagement approaches, between Kilifi (Coastal Kenya) and Ahero (Western Kenya). Some similarities included large community meetings, while differences included working with community health volunteers in engagement, which was done in Ahero but not in Kilifi. In addition, in Kilifi, field staff training on communication and consenting forms part of engagement activities. Field workers are often the face of the institution in the community and encounter challenging questions about research being conducted by the organization, as they visit homes to give study information and refer potential volunteers for screening. As such, the CHMI–SIKA field workers went through a communications and consent training before carrying out study activities, as is done for all studies undertaken in the Programme. In addition, the field workers had extensive training including role plays on how to effectively communicate the key messages of the study. This allowed for an internal evaluation and feedback based on the role plays conducted. The role plays involved selected members of the field team acting as potential volunteers who would be approached for information giving about the study with feedback on how the information was relayed and whether reflective of the key messages. The key messages document was useful in ensuring that during the training, field workers understood how to frame messages around the study uniqueness, thereby, ensuring that what they said was consistent with the information shared through engagement activities. Based on sentiments shared by community members in routine engagement activities, consistency in messaging is a key marker for trust. Conflicting messages coming from members of the same institution are considered to be a flag for dishonesty with the potential for breaking trust.

Throughout the CHMI–SIKA study, the community engagement and study teams worked together to address a range of challenges. For example, engagement activities were conducted around April/May, which in the Coastal part of Kenya, is the long rainy period. Often, meetings had to be postponed either due to heavy rains or because community members were busy in their farms. The CHMI–SIKA study team appreciated these challenges and were willing to be flexible. However, the CLG staff understood the tight study timelines and made efforts to negotiate with community leaders to continue with some of the meetings through sourcing for in-door venues where residents would be sheltered from the rain. In planning for community engagement activities, research teams must be cognizant of community socio-cultural norms and practices, as these can sometimes have a direct impact on engagement, recruitment, and study timelines.

Another challenge that faced the CHMI–SIKA study was that after the first cohort of participants had been successfully enrolled and completed follow-up with the publishing of the embedded social science study (4), a news article was published in a widely read national newspaper, that both overstated the risks of HIS for falciparum malaria and the levels of compensation provided to participants (23). Interestingly, the journalist who wrote this article had attended a workshop set up by the public engagement team at KWTRP and gathered information about this novel research approach from the study team present during the workshop. A communication piece had also previously been shared with the national newspaper editorial team before the study started. Perhaps even more interesting, the main public response received was a high level of enquiries about opportunities for participating in studies like this, rather than criticism around the safety of research being conducted. An important lesson to learn here is that despite engaging with journalists, there might be one or two who develop unexpected lines of reporting that the engagement and communications team has no control over. This can be due to the interests of the media not being aligned with those of the investigators. In our case, we responded to the article published through a press statement, which was posted on our institution's social media account (Twitter).

Determining engagement effectiveness is a complex task that involves having first outlined goals and objectives for evaluation. However, we think that our engagement was useful in some ways as the CHMI–SIKA study was conducted successfully from beginning to end without major interruptions. KWTRP's long-standing relationship with the community members in the KHDSS might have helped make engagement sessions smoother. The community is aware and expects that every new study recruiting human volunteers will be brought to a community meeting for dialogue before the study commences. This has helped to build trust with this community, which is critical when conducting research such as HIS.

In addition, the engagement process as well as on-going study interactions and embedded social science studies helped to identify key areas of concerns early on in the study, which helped in addressing these and initial fears, questions about the research design, safety concerns (including potential for third party risks which this study did not present). This we think might have contributed to allaying initial fears and hesitation among potential volunteers.

Administering informed consent took account of views from the community; it was a process with several interactions with study team members, and extended time for discussions with family members, as was requested by community members in engagement activities (4). We postulate that these multiple engagement processes made information accessible to potential volunteers because they had some level of information obtained either from the community meeting, or a friend who had attended a CHMI–SIKA engagement meeting or had been a study volunteer.

Following successful completion of the first cohort in 2016 which enrolled 37 volunteers, the majority of these volunteers became self-appointed “study ambassadors” and communicated their experiences of participation as well as information about the study in the community. Taken together, the initiation of in-residence workshops (that allowed for close cohort-specific volunteer engagement with research) and embedded social science and empirical ethics work, allowed for a volunteer-centered engagement approach for direct feedback into the processes and procedures of the study conduct. This for instance resulted in a better understanding of the need to stay in residence. There was a co-adaption of learnings from each engagement process from one cohort to the other.

## Conclusion

Despite HIS being relatively new in Kenya, the high-level stakeholder engagement meetings held in Nairobi during the very first “challenge” study paved the way for successfully carrying out the study in Kilifi. In addition, conducting a broad range of engagement activities, right from the protocol development stage to the formation of a CAST, developing key messages and using these for consistent and correct messaging during engagement implementation, minimized the chances of raising rumors about the study. These approaches also provided a forum where multiple stakeholders raised concerns and questions related to CHMI–SIKA and HIS in general and had these responded to. Research and engagement teams can draw on these approaches including lessons that have been learnt as a reference for future HIS engagement planning and implementation.

## Data Availability Statement

The original contributions presented in the study are included in the article/supplementary material, further inquiries can be directed to the corresponding author.

## Author Contributions

NM, PN, PB, DK, and MK contributed to the conception and design of the work. NM, PN, CM, SM, JM, MM, BK, HA, JW, PB, and MK conducted engagement activities. NM wrote the first draft of the manuscript. MK, DK, PB, MH, VM, EA, CM, and PC contributed to manuscript revision, read, and approved the submitted version. All authors contributed to the article and approved the submitted version.

## Funding

This work was funded by the Wellcome Trust (Grant Number: 107499). The funder had no role in the design, analysis, write up, or decision to submit for publication.

## Conflict of Interest

The authors declare that the research was conducted in the absence of any commercial or financial relationships that could be construed as a potential conflict of interest.

## Publisher's Note

All claims expressed in this article are solely those of the authors and do not necessarily represent those of their affiliated organizations, or those of the publisher, the editors and the reviewers. Any product that may be evaluated in this article, or claim that may be made by its manufacturer, is not guaranteed or endorsed by the publisher.
